# ADULT CHILDREN AND THEIR BOOMER PARENTS: THE DYNAMICS OF INTERGENERATIONAL LIVING ARRANGEMENT AND LIFE EVENTS

**DOI:** 10.1093/geroni/igz038.548

**Published:** 2019-11-08

**Authors:** Chiu-hua Huang, Ju-ping Lin

**Affiliations:** 1 Shih Chien University, Taipei, Taiwan; 2 National Taiwan Normal University, Taipei, Taiwan

## Abstract

Taiwan became an aged society in 2018. As Baby Boomers enter late life, relationships with family members gain importance.This research aimed to examine the intergenerational living arrangement between adult children and their baby boomer parents. Taking the perspectives of adult children, five waves of data (2008, 2009, 2010, 2011, and 2012) from the Panel Study of Family Dynamics (PSFD) were analyzed. Latent class growth analysis (LCGA) was used to develop changes types of intergenerational living arrangements. Focus on the life events of the two generations, this research examined the effect on intergenerational living arrangements changes pattern. The main findings are as follow. First, The proportion of intergenerational co-residence is high, especially between adult sons and their parents. There are different types of changes of intergenerational living arrangements. The four types of changes of intergenerational living arrangements for adult sons and daughters are the same: “continuous co-residence,” “continuous non-co-residence,” “from co-residence to non-co-residence,” and “from non-co-residence to co-residence.” Second, Adult children’s life events such as getting married and having children affect changes of intergenerational living arrangements. After marrying, the intergenerational living arrangement between adult children and their parents is inclined to be the “continuous non- co-residence” type. When adult sons have newborn babies, the living arrangement is inclined to be “from co-residence to non-co-residence.”

